# Supplementary material for: Asymmetric reinforcement learning facilitates human inference of transitive relations
Source: Nat Hum Behav. 2022 Jan 31;6(4):555–64. doi: 10.1038/s41562-021-01263-w (PMC9038534; doi:10.1038/s41562-021-01263-w)
Supplement: Supplementary file 1 — Supplementary Tables 1–4 and Methods. [file 41562_2021_1263_MOESM1_ESM.pdf]

nature portfolio

https://doi.org/10.1038/s41562-021-01263-w

Supplementary information

Asymmetric reinforcement learning facilitates human inference of transitive relations

In the format provided by the authors and unedited

Asymmetric reinforcement learning facilitates human inference of transitive relations

Simon Ciranka[1,2]*, Juan Linde-Domingo[1]*, Ivan Padezhki[1], Clara Wicharz[1], Charley M. Wu[1,3], and Bernhard Spitzer[1,2]**

Supplementary Information

[1]Center for Adaptive Rationality, Max Planck Institute for Human Development, Berlin, Germany

[2]Max Planck UCL Centre for Computational Psychiatry and Ageing Research, Berlin, Germany

[3]Human and Machine Cognition Lab, University of Tübingen, Tübingen, Germany

*These authors contributed equally
**corresponding author, email: spitzer@mpib-berlin.mpg.de

Supplementary Tables

Supplementary Table 1. Tests of mean accuracy on non-neighbour trials against chance (Wilcoxon signed-rank tests against 0.5) in the individual experiments with partial feedback.

	z	n	r	95% CI	p-value
Experiment 2	-4.68	31	0.84	[0.75,0.87]	<.001
Experiment 3	-6.00	48	0.87	[0.85, 0.87]	<.001
Experiment 4	-6.08	49	0.87	[0.87,0.87]	<.001

Supplementary Table 2. Fit of symmetric models (Q1, Q1*, Q1*+P, Q1*+Pi) tested against their asymmetric counterparts (Wilcoxon signed-rank tests comparing BICs, aggregated across Experiments 2-4 with partial feedback).

	z	n	r	95% CI	p-value
Q1 vs. Q2	-4.06	128	0.36	[0.19, 0.50]	<.001
Q1* vs. Q2*	-8.53	128	0.75	[0.67, 0.81]	<.001
Q1*+P vs. Q2*+P	-7.79	128	0.69	[0.59, 0.76]	<.001
Q1*+Pi vs. Q2*+Pi	-7.08	128	0.63	[0.52, 0.72]	<.001

Supplementary Table 3. Fit of symmetric versus asymmetric models (Wilcoxon signed-rank tests comparing BICs) in the individual experiments with partial feedback.

	z	n	r	95% CI	p-value
Experiment 2	-4.67	31	0.76	[0.57, 0.85]	<.001
Experiment 3	-5.02	48	0.67	[0.49, 0.80]	<.001
Experiment 4	-5.37	49	0.70	[0.54,0.81]	<.001

Supplementary Table 4. Fit of previously proposed models compared to our winning model Q2*+P (Wilcoxon signed-rank tests comparing BICs, aggregated across Experiments 2-4).

	z	n	r	95% CI	p-value
VAT	-7.40	128	0.65	[0.55, 0.74]	<.001
RL-ELO	-8.70	128	0.76	[0.70, 0.82]	<.001
VAT2+P	-2.45	128	0.22	[0.06,0.39]	.014
RL-ELO2+P	-3.73	128	0.33	[0.16,0.48]	<.001

Supplementary Methods

RL-ELO

When fitting RL-ELO, we replaced our Q-learning process (*Methods*: *Item-level learning, Eq. 1*) by a rank learning process as proposed by Kumaran and colleagues[1]

$$V_{t+1}(i) \;=\; V_t(i) \;+\; \alpha\big[\;\;1 - CP_{win,t}\big]$$
$$V_{t+1}(j) \;=\; V_t(j) \;+\; \alpha\big[-1 + CP_{win,t}\big]$$

where $V(i)$ and $V(j)$ are the ranks of the winning item i and the losing item j, CP_{win} is the probability of choosing the winning item, and α is the learning rate. CP_{win} was computed with a logistic choice function (analogous to Eq. 5) of the difference in ranks between the winning and the losing item $[V(i) - V(j)]$.

Value-transfer

The value transfer model (VAT) proposed by von Fersen and colleagues[2] assumes that the value of the losing item is updated with a proportion of the value of the winning item. We implemented VAT in a similar form as described previously[1]:

$$V_{t+1}(i) \;=\; V_t(i) \;+\; \alpha[\;\;1 - V_t(i)]$$
$$V_{t+1}(j) \;=\; V_t(j) \;+\; \alpha[-1 - V_t(j)] + V_t(i) * \theta$$

where $V(i)$ and $V(j)$ are the values of the winning item i and the losing item j, α is the learning rate, and θ controls the value transfer from the winning to the losing item. Interestingly, this formulation of VAT incorporates a form of asymmetric learning (through value transfer from winner to loser but not vice versa), and it can even predict below-chance performance for certain item pairings (through exceedingly large values of θ), similar to our Q2* model family. However, the Q2* process provided a better description of our empirical data (see *Results*).

For comparisons with our winning model (Q2*+P), we additionally fitted extended variants of RL-ELO and VAT where we included separate learning rates for winner and losers (α^+ and α^-, analogous to our model Q2, see *Methods*, equation 3) as well as pair-level learning (+P, equations 6-7 and 9-10).

References

1. Kumaran, D., Banino, A., Blundell, C., Hassabis, D. & Dayan, P. Computations Underlying Social Hierarchy Learning: Distinct Neural Mechanisms for Updating and Representing Self-Relevant Information. *Neuron* **92**, 1135–1147 (2016).

2. von Fersen, L., Wynne, C. D., Delius, J. D. & Staddon, J. E. Transitive inference formation in pigeons. *Journal of Experimental Psychology: Animal Behavior Processes* **17**, 334–341 (1991).
