# Supplementary material for: Asymmetric reinforcement learning facilitates human inference of transitive relations
Source: Nat Hum Behav. 2022 Jan 31;6(4):555–64. doi: 10.1038/s41562-021-01263-w (PMC9038534; doi:10.1038/s41562-021-01263-w)
Supplement: Supplementary file 2 — Reporting Summary. [file 41562_2021_1263_MOESM2_ESM.pdf]

nature research

Corresponding author(s): Bernhard Spitzer

Last updated by author(s): Oct 11, 2021

Reporting Summary

Nature Research wishes to improve the reproducibility of the work that we publish. This form provides structure for consistency and transparency in reporting. For further information on Nature Research policies, see our Editorial Policies and the Editorial Policy Checklist.

Statistics

For all statistical analyses, confirm that the following items are present in the figure legend, table legend, main text, or Methods section.

n/a	Confirmed	
☐	☒	The exact sample size (*n*) for each experimental group/condition, given as a discrete number and unit of measurement
☐	☒	A statement on whether measurements were taken from distinct samples or whether the same sample was measured repeatedly
☐	☒	The statistical test(s) used AND whether they are one- or two-sided<br>*Only common tests should be described solely by name; describe more complex techniques in the Methods section.*
☒	☐	A description of all covariates tested
☐	☒	A description of any assumptions or corrections, such as tests of normality and adjustment for multiple comparisons
☐	☒	A full description of the statistical parameters including central tendency (e.g. means) or other basic estimates (e.g. regression coefficient) AND variation (e.g. standard deviation) or associated estimates of uncertainty (e.g. confidence intervals)
☐	☒	For null hypothesis testing, the test statistic (e.g. *F*, *t*, *r*) with confidence intervals, effect sizes, degrees of freedom and *P* value noted<br>*Give P values as exact values whenever suitable.*
☒	☐	For Bayesian analysis, information on the choice of priors and Markov chain Monte Carlo settings
☒	☐	For hierarchical and complex designs, identification of the appropriate level for tests and full reporting of outcomes
☐	☒	Estimates of effect sizes (e.g. Cohen's *d*, Pearson's *r*), indicating how they were calculated

Our web collection on statistics for biologists contains articles on many of the points above.

Software and code

Policy information about availability of computer code

Data collection	Exp. 1-2: Matlab R2017a (MathWorks), Psychophysics Toolbox Version 3 (Brainard, 1997); Exp. 3-4: Psychopy (https://www.psychopy.org/builder/builder.html); custom code: https://doi.org/10.5281/zenodo.5561411
Data analysis	R (http://www.R-project.org/), Matlab R2020a (MathWorks); custom code: https://doi.org/10.5281/zenodo.5561411

For manuscripts utilizing custom algorithms or software that are central to the research but not yet described in published literature, software must be made available to editors and reviewers. We strongly encourage code deposition in a community repository (e.g. GitHub). See the Nature Research guidelines for submitting code & software for further information.

Data

Policy information about availability of data

All manuscripts must include a data availability statement. This statement should provide the following information, where applicable:

- Accession codes, unique identifiers, or web links for publicly available datasets
- A list of figures that have associated raw data
- A description of any restrictions on data availability

The data that support the findings of this study are available at: https://doi.org/10.5281/zenodo.5561411

Field-specific reporting

Please select the one below that is the best fit for your research. If you are not sure, read the appropriate sections before making your selection.

☐ Life sciences ☒ Behavioural & social sciences ☐ Ecological, evolutionary & environmental sciences

For a reference copy of the document with all sections, see nature.com/documents/nr-reporting-summary-flat.pdf

Behavioural & social sciences study design

All studies must disclose on these points even when the disclosure is negative.

Study description	Quantitative experimental (four experiments) and computational modeling /simulation
Research sample	Participants in Exp. 1 and 2 were healthy young adults recruited from a participant pool at the Max Planck Institute for Human Development in Berlin, Germany. Of these, n=20 participated in Experiment 1, (13 female, 7 male, mean age 27.15 ± 3.91 years) and n=35 participated in Exp. 2 (14 female, 27 ± 3.80 years). Participants in Exp. 3 and 4 were healthy young adults recruited online via Prolific Academic (https://www.prolific.co) with n=76 completing Exp. 3 (23 female, 24.73 ± 5.40 years) and n=60 completing Exp. 4 (23 female; 25.92 ± 4.54 years). Informed consent was obtained by all participants. The online samples were restricted to English speaking participants.
Sampling strategy	Participants were assigned to the different experiments by opportunity sampling. A pilot experiment with partial feedback (cf. supplementary information) suggested that learning asymmetries could be detected in a sample of n=11 participants (p<0.05, comparison of model BICs), which was substantially exceeded in all experiments (1-4). Sampling sizes in the online experiments (3-4) were chosen to be larger than in the in-lab experiments (1-2) due to anticipated higher drop-out rates in online testing.
Data collection	Experiments 1 and 2 were conducted in lab, using Psychophysics Toolbox Version 3 (Brainard, 1997) running in MATLAB 2017a. In Exp. 2, we additionally recorded electroencephalography for the purpose of a different research question. The experimenters present during in-lab testing were blind to the object-value associations. Experiments 3 and 4 were conducted online (on https://www.pavlovia.org), using Qualtrics (https://www.qualtrics.com) for statement of consent and basic demographics.
Timing	Start and finish of data collection:<br>Exp. 1 from 11th January 2019 to 15th January 2019<br>Exp. 2 from 13th March 2019 to 5th July 2019 (n=28). A second cohort (n=7) was collected for the purpose of EEG recordings from 9th March 2020 to 4th September 2020 (with forced breaks due to COVID-19 pandemic) .<br>Exp. 3 from 1st July 2020 to 4th July 2020.<br>Exp. 4 from 30th September 2020 to 1st October 2020.
Data exclusions	The data from participants who did not reach above-chance learning levels were excluded from analysis. The threshold for inclusion was set to 60% correct judgments in the last two blocks of the experiment, which corresponds to a binomial test probability of p<0.01 (uncorrected) compared to chance-level (50%). After exclusion, n=17 (Exp. 1), n=31 (Exp. 2), n=48 (Exp. 3) and n=49 (Exp. 4) participants remained for analysis.
Non-participation	No participants dropped out or declined participation in Exp. 1-2. In Exp. 3-4 (online studies), n=12 (Exp. 3) and n=8 (Exp.4) individuals signed up but did not complete the experiment.
Randomization	Participants were randomly assigned to the individual experiments (opportunity sampling). The association between stimuli and its value structure was pseudo-randomly assigned to the pictures for each participant. Stimulus pairings (8 neighbors and 20 non-neighbors) were randomly intermixed across trials, with randomized ordering of the elements in a pair (e.g., A-B or B-A).

Reporting for specific materials, systems and methods

We require information from authors about some types of materials, experimental systems and methods used in many studies. Here, indicate whether each material, system or method listed is relevant to your study. If you are not sure if a list item applies to your research, read the appropriate section before selecting a response.

Materials & experimental systems

n/a	Involved in the study	
☒	☐	Antibodies
☒	☐	Eukaryotic cell lines
☒	☐	Palaeontology and archaeology
☒	☐	Animals and other organisms
☐	☒	Human research participants
☒	☐	Clinical data
☒	☐	Dual use research of concern

Methods

n/a	Involved in the study	
☒	☐	ChIP-seq
☒	☐	Flow cytometry
☒	☐	MRI-based neuroimaging

Human research participants

Policy information about studies involving human research participants

Population characteristics	Young healthy adults.
Recruitment	In Exp. 1 and 2, participants were recruited from a participant pool at the Max Planck Institute for Human Development. Participants in Exp. 3 and 4 were recruited online via Prolific Academic (www.prolific.co).
Ethics oversight	All experiments were approved by the ethics committee of the Max Planck Institute for Human Development and conducted in accordance with the Human Subjects Guidelines of the Declaration of Helsinki.

Note that full information on the approval of the study protocol must also be provided in the manuscript.
