# Supplementary material for: Asymmetric reinforcement learning facilitates human inference of transitive relations
Source: Nat Hum Behav. 2022 Jan 31;6(4):555–64. doi: 10.1038/s41562-021-01263-w (PMC9038534; doi:10.1038/s41562-021-01263-w)
Supplement: Supplementary file 4 — Peer Review Information. [file 41562_2021_1263_MOESM4_ESM.pdf]

natureresearch

Peer Review Information

Journal: Nature Human Behaviour
Manuscript Title: Asymmetric reinforcement learning facilitates human inference of transitive relations
Corresponding author name(s): Bernhard Spitzer

Editorial Notes:

**Redactions – unpublished data**	Parts of this Peer Review File have been redacted as indicated to maintain the confidentiality of unpublished data.

Reviewer Comments & Decisions:

Decision Letter, initial version:

17th May 2021

Dear Dr Spitzer,

Thank you once again for your manuscript, entitled "Asymmetric learning facilitates human inference of transitive relations", and for your patience during the peer review process.

Your Article has now been evaluated by 2 referees. You will see from their comments copied below that, although they find your work of considerable potential interest, they have raised quite substantial concerns. In light of these comments, we cannot accept the manuscript for publication, but would be interested in considering a revised version if you are willing and able to fully address reviewer and editorial concerns.

We hope you will find the referees' comments useful as you decide how to proceed. If you wish to submit a substantially revised manuscript, please bear in mind that we will be reluctant to approach the referees again in the absence of major revisions. We are committed to providing a fair and constructive peer-review process. Do not hesitate to contact us if there are specific requests from the reviewers that you believe are technically impossible or unlikely to yield a meaningful outcome.

We consider two issues key in revision. First, we ask you to provide further model comparisons and validation of the present models in response to the comments made by Reviewers #1 and #2. Second, we ask you to provide a clearer and more comprehensive presentation of the human data as

requested by Reviewer #2, and relatedly general improvements to the accessibility of the presentation of the work (Reviewer #1).

Finally, your revised manuscript must comply fully with our editorial policies and formatting requirements. Failure to do so will result in your manuscript being returned to you, which will delay its consideration. To assist you in this process, I have attached a checklist that lists all of our requirements. If you have any questions about any of our policies or formatting, please don't hesitate to contact me.

If you wish to submit a suitably revised manuscript we would hope to receive it within 6 months. We understand that the COVID-19 pandemic is causing significant disruptions which may prevent you from carrying out the additional work required for resubmission of your manuscript within this timeframe. If you are unable to submit your revised manuscript within 6 months, please let us know. We will be happy to extend the submission date to enable you to complete your work on the revision.

With your revision, please:

• Include a “Response to the editors and reviewers” document detailing, point-by-point, how you addressed each editor and referee comment. If no action was taken to address a point, you must provide a compelling argument. This response will be used by the editors to evaluate your revision and sent back to the reviewers along with the revised manuscript.

• Highlight all changes made to your manuscript or provide us with a version that tracks changes.

Please use the link below to submit your revised manuscript and related files:

[REDACTED]

Note: This URL links to your confidential home page and associated information about manuscripts you may have submitted, or that you are reviewing for us. If you wish to forward this email to co-authors, please delete the link to your homepage.

Thank you for the opportunity to review your work. Please do not hesitate to contact me if you have any questions or would like to discuss the required revisions further.

Sincerely,

Marike

Marike Schiffer, PhD
Senior Editor
Nature Human Behaviour

Reviewer expertise:

Reviewer #1: decision making, computational modelling

Reviewer #2: decision making, computational modelling

REVIEWER COMMENTS:

Reviewer #1:
Remarks to the Author:
In this manuscript, Ciranka, Linde-Domingo and colleagues propose a simple reinforcement learning (RL) mechanism to solve transitive inference (e.g. learning that A>C from A>B and B>C). First, using computational simulations, they show that specific classes of RL models can achieve transitive inferences when experiencing feedback from all pairs, or just neighboring pairs (i.e. A<>B and B<>C). Then, over a series of behavioral experiments in human participants, they show that the pattern of behavior is consistent with their proposed model.

Overall, I found the topic stimulating and the manuscript thought-provoking. The combination of model simulation, behavioral experiments & model fitting is state of the art. The analytical approach is quite exhaustive/thorough and sophisticated – sometimes even clever (e.g. I liked the dissociation between p(fit¦gen) and p(gen¦fit) in the model identification exercise – Fig S5; will use it myself in the future). This unfortunately occasionally comes at the cost of clarity: I found some sections quite dense (Methods section on Pair-relational learning), and some are quite elusive (digressions on below-chance accuracy and negative alpha_minus). I suggest that some writing work is done during the revision, to make sure that all sections are fully understandable to a naïve but interested reader.

I only have a couple of general comments and minor suggestions which I hope the authors will find useful.

Major:

Although I'm never a big fan of reviewers asking to integrate/test more models, I'm a bit puzzled by the fact that all models proposed by the authors are completely agnostic to participants choices, as they only use the feedback to update the value regardless of the choice/correctness. I see two dimensions in which that might be an issue.

- First, it is well known that choices interact with feedback in the way this latter is integrated into learning signals; (refs 23-28 in the authors manuscript). Thereby, neglecting this dimension might miss some behavioral patterns – and conversely, integrating this dimension on top of the current model might provide a better fit to participants' data. For instance it struck me that behavioral patterns shown in Fig 3ab and Fig.4 do not seem to show the value compression that is supposed to be a signature of the Q2* model. I'm wondering whether integrating some confirmatory learning on top of the authors' current models could mitigate this issue and/or improve the general fits.

- Second, I found the Pair-relational learning mechanism quite circumvoluted, and am wondering whether a simple Actor-Critic-like architecture would (more) simply make similar predictions, by reinforcing the policy of selecting the high value item in the specific state where the action is reinforced (i.e. neighboring pairs), in addition to updating its value (for all transitive inferences).

Minor:

- I would appreciate a (supplementary) figure depicting the (distribution of) parameters fitted in the different experiments.

- As I said earlier, I find the model identification quite elegant, but there seem to be some issues with the Fig S5
p(fit¦gen) as some columns do not seem to sum to 1.

- I am wondering why learning rates in simulations and fitting are restricted to such narrow ranges (traditionally, in RL, they can span from 0 to 1).

- Figure S4 I suggest to use a single colorbar (i.e. same y-axis) for the different panels, to visually appreciate the modulation of general performance due to decision noise

- Figure 4 right: It would be nice to find a visual "trick" to highlight above versus below chance levels (e.g. a disjointed color scale centered on 50?)

- There is no y-axis to index BIC levels on Figs 3 e-f

Reviewer #2:
Remarks to the Author:

This study introduces a novel behavioral paradigm for the learning of abstract serial order. It then shows that reinforcement learning algorithms with asymmetric updating rules can provide an account of human performance in this paradigm. These are notable contributions to the field. There are some omissions and inconsistencies that should be addressed. The manner in which the data are reported is quite frustrating. The human data are simply reported as a summary statistic presumably reflecting average performance of all subjects over some time interval. There is little information about between-subject variation in learning rate or asymptotic performance. This makes it difficult for the reader to appreciate how well the various models capture important aspects of the data.

General comments:

Partial feedback, as applied to transitive inference, appears to be a novel behavioral paradigm with some intriguing properties. It has been used before, but usually in a transfer paradigm and not a situation where subjects are simultaneously training on adjacent pairs and being tested on non-adjacent pairs. This has the benefit of allowing one to compare learning for adjacent and non-adjacent pairs in parallel. Unfortunately, the dynamics of learning (e.g. performance vs. trial number) are not reported for the human subjects. This is a significant omission for a paper about learning, and deprives the reader of being able to really appreciate the richness of the data.

Asymmetric Q-learning is a somewhat novel modeling approach. However, the issue was discussed in Jensen et al., 2019 (see reference below), which may deserve some mention.

It would be worth discussing the issue of stability over time. In particular, the Q2 model does not

appear to be asymptotically stable. Rather, it appears that all of the values would eventually saturate at 1.0 given enough trials. Q2*, on the other hand, does appear to be stable. This is an important consideration for model fitting and selection. It would seem difficult to fit an unstable model without making strong assumptions about learning rates. Furthermore, if the values of all items in the model do indeed saturate, then it is hard to see how this could be a viable model of TI, as it would eventually lose the ability to support non-random choices between pairs of items.

The dynamics of learning are shown for the models, but not for the human data. Regarding the latter, it isn't clear if the data shown in Figs 3 and 4 are averaged over the entire session or reflect asymptotic performance. Likewise, it isn't clear if the models are fit to the entire time course of the data or to asymptotic performance. A figure that shows model and real performance as a function of trial number would help readers evaluate how well the models actually fit the data.

Specific comments

Figures – The color-coded half grids provide a general sense of the results and are fine for showing the model predictions (Fig. 2). However, for empirical data, this style of presentation makes it difficult for readers to see more subtle effects, make quantitative comparisons, or get a sense of the variability in the data. For figures 3 and 4, it might be advisable to use more conventional "box and whisker" style plotting.

Likewise, the stacked histograms in Fig 4 (middle panel) are difficult to decipher. There seems to be enough space to separate these into 4 separate subpanels with one histogram each. This would be greatly appreciated.

Models Q2 and Q2* make the strong prediction that performance should depend on absolute rank even for pairs that have the same symbolic distance. I.e., for distance=1, the pair AB should have the worst performance, while GH should have the best performance. This is borne out by the data only in the case of GH. All of the other pairs that have distance=1 appear to have about the same performance level. In other words, the strong vertical gradient shown for Q2 and Q2* in Fig. 2 does not appear to be supported by the data in Fig. 3. Furthermore, it is not clear why the model predictions for Q2* appear to be quite different in Fig. 2 vs. Fig. 3. Perhaps the authors could address the discrepancy.

References

Jensen G, Terrace HS, Ferrera VP. Discovering Implied Serial Order Through Model-Free and Model-Based Learning. Front Neurosci. 2019 Aug 20;13:878. doi: 10.3389/fnins.2019.00878. PMID: 31481871; PMCID: PMC6710392.

Author Rebuttal to Initial comments

Response to the Reviews

(original comments in *italic*)

EDITOR COMMENTS

We consider two issues key in revision. First, we ask you to provide further model comparisons and validation of the present models in response to the comments made by Reviewers #1 and #2. Second, we ask you to provide a clearer and more comprehensive presentation of the human data as requested by Reviewer #2, and relatedly general improvements to the accessibility of the presentation of the work (Reviewer #1).

We wish to thank the editor very much for inviting us to submit a revision. We believe that we were able to address all these aspects in full, as outlined in our detailed response to the reviews below. To summarize the key elements of the revision:
(i) We included additional model comparisons and further analyses requested by the reviewers, all of which corroborated and validated our previous findings.
(ii) We substantially expanded the presentation of the human data, including a comprehensive new figure (Fig. 4 in the revised manuscript) showing the time-course of learning, inter-subject variability, and additional features of the data as requested by Reviewer #2.
(iii) We rewrote large parts of the manuscript to increase clarity, and carefully edited the manuscript throughout, with particular attention to the aspects highlighted by Reviewer #1.

We believe that the revisions strengthened our paper considerably. Please see our detailed replies to the individual reviewer comments below.

In addition, we reformatted the manuscript according to the journal guidelines for final submission, as requested in the action letter. Newly added sections are marked in blue color.

REVIEWER COMMENTS:

Reviewer #1:
Remarks to the Author:
In this manuscript, Ciranka, Linde-Domingo and colleagues propose a simple reinforcement learning (RL) mechanism to solve transitive inference (e.g. learning that A>C from A>B and B>C). First, using computational simulations, they show that specific classes of RL models can achieve transitive inferences when experiencing feedback from all pairs, or just neighboring pairs (i.e. A<>B and B<>C). Then, over a series of behavioral experiments in human participants, they show that the pattern of behavior is consistent with their proposed model.

Overall, I found the topic stimulating and the manuscript thought-provoking. The combination of model simulation, behavioral experiments & model fitting is state of the art. The analytical approach is quite exhaustive/thorough and sophisticated – sometimes even clever (e.g. I liked the dissociation between p(fit|gen) and p(gen|fit) in the model identification exercise – Fig S5; will use it myself in the future). This unfortunately occasionally comes at the cost of clarity: I found some sections quite dense (Methods section on Pair-relational learning), and some are quite elusive (digressions on below-chance accuracy and negative alpha_minus). I suggest that some writing work is done during the revision, to make sure that all sections are fully understandable to a naïve but interested reader.

We thank the reviewer for their positive evaluation of our manuscript. We agree that owing to the study's technical complexion, some sections appeared rather dense. Thus, we took great effort in the revision to improve the accessibility of the paper. Specifically, we rewrote the sections on pair-level learning (both in the main text and in the Methods section; p 8 and p 26-27) and on below-chance accuracies (p 17), in addition to various edits for increased clarity throughout the entire manuscript. We hope the reviewer finds these sections now presented in a more accessible manner.

In addition, we included a schematic visualisation of our model space in supplementary S1 (new panel c) to provide further assistance for interested readers. Please also see our "Further Revisions" below, which also add to the accessibility of our results report. We wish to thank the reviewer for the helpful comment.

I only have a couple of general comments and minor suggestions which I hope the authors will find useful.

Major:

Although I'm never a big fan of reviewers asking to integrate/test more models, I'm a bit puzzled by the fact that all models proposed by the authors are completely agnostic to participants choices, as they only use the feedback to update the value regardless of the choice/correctness. I see two dimensions in which that might be an issue.
- First, it is well known that choices interact with feedback in the way this latter is integrated into learning signals; (refs 23-28 in the authors manuscript). Thereby, neglecting this dimension might miss some behavioral patterns – and conversely, integrating this dimension on top of the current model might provide a better fit to participants' data. For instance it struck me that behavioral patterns shown in Fig 3ab and Fig.4 do not seem to show the value compression that is supposed to be a signature of the Q2 model. I'm wondering whether integrating some confirmatory learning on top of the authors' current models could mitigate this issue and/or improve the general fits.*

The reviewer correctly points out that the feedback learning in our models was agnostic to participants' individual choices. In this respect, our models are in the tradition of previous

models of transitive inference (e.g., refs. [14,21,22] in the manuscript; see ref. [37] for exceptions). In the revised manuscript, we extended our models in several additional ways to also incorporate trial-by-trial participant behaviour. First, (see also comments by Reviewer #2), we tested whether our results may alternatively be explained by an asymmetry between chosen/unchosen items, (cf. ref. [37]), rather than by the winner/loser asymmetries highlighted in our paper. We found this not to be the case (p 14), which strengthens our previous conclusions.

Second, as suggested by the reviewer, we extended our models to allow for differential learning from confirmatory/disconfirmatory choice feedback. Indeed, this extension further improved the fit of our model (p14, on bottom), consistent with previous work in other learning contexts (refs. [24,25,28]). However, the model extension did not alter our findings about winner/loser asymmetries: we obtained equivalent results with regards to winner/loser asymmetries, regardless of whether the model extension was included (see new supplementary Fig. S5) or not (cf. Fig. 3f). We thank the reviewer for encouraging us to explore these additional models, which further validate our findings, and which improve the linkage to previous literatures in other learning domains. Addressing the reviewer's comment on the behavioural signatures of value compression, please see our reply to the last comment by Reviewer #2 below, where we address this point in detail.

- *Second, I found the Pair-relational learning mechanism quite circumvoluted, and am wondering whether a simple Actor-Critic-like architecture would (more) simply make similar predictions, by reinforcing the policy of selecting the high value item in the specific state where the action is reinforced (i.e. neighboring pairs),* in addition to updating its value *(for all transitive inferences).*

We thank the reviewer for this feedback, which has helped us improve the clarity and interpretability of the paper. In revising the manuscript, we paid particular attention to explaining the logic behind pair-level learning more clearly. In fact, we think that our basic pair-level learning (+P) reflects a simplistic learning process quite similar to what the reviewer suggests: When experiencing a neighbour comparison (e.g., B-C), participants may learn to provide a certain response (e.g., B). Likewise, for another neighbour comparison (e.g., C-D), they may learn a different response (e.g., C). We believe that in its simplicity, this process does encapsulate a basic stimulus-action reinforcement of the kind the reviewer mentioned. We thoroughly revised the presentation of pair-level learning to convey this more clearly (main text: p 8; Methods: p 26-27; see also new Supplementary Fig. S1c).

Minor:

- *I would appreciate a (supplementary) figure depicting the (distribution of) parameters fitted in the different experiments.*

We thank the reviewer for this suggestion. In the revised manuscript, we include a supplementary figure (new supplementary Fig. S6) illustrating the parameter estimates for every participant in each experiment.

- As I said earlier, I find the model identification quite elegant, but there seem to be some issues with the Fig S5 p(fit|gen) as some columns do not seem to sum to 1.

We thank the reviewer for this detailed level of feedback. We verified that these small deviations reflect rounding imprecision. We now clarify this information in the revised figure caption (now Fig. S7 in the revised supplement).

- I am wondering why learning rates in simulations and fitting are restricted to such narrow ranges (traditionally, in RL, they can span from 0 to 1).

The parameter ranges were derived from extensive simulations of our relational learning tasks, which differ in several respects from more traditional RL tasks. Learning rates larger than 0.2 produce erratic learning patterns and near-chance performance in our tasks. Consistently, larger learning rates also suffer from poor recoverability when fitting simulated data (see supplementary Fig. S10). Restricting the range of parameter values to sensible learning rates (in the context of our present experiments) improves the stability of our fits and fosters better interpretability of the results.

- Figure S4 I suggest to use a single colorbar (i.e. same y-axis) for the different panels, to visually appreciate the modulation of general performance due to decision noise

We share this thought. However, we found that constant color scaling makes it difficult to discern the critical patterns within each panel, due to the large differences in overall performance levels. We include below an alternative figure with constant color scaling for the reviewer to verify. To better highlight the general performance levels in our supplementary Figure S4, we added a note on the different color bars in the revised figure caption.

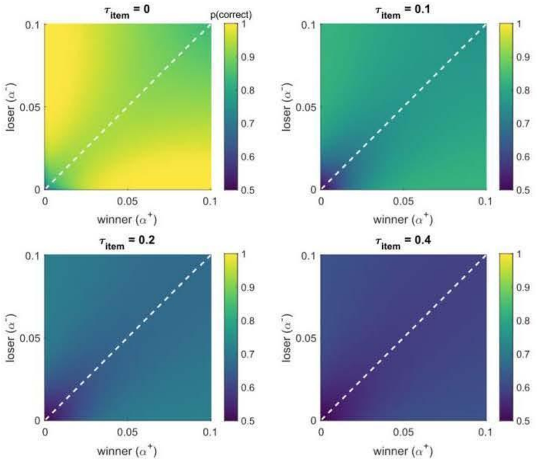

- Figure 4 right: It would be nice to find a visual "trick" to highlight above versus below chance levels (e.g. a disjointed color scale centered on 50?)

We agree, this is another great suggestion. We added markers (white crosses) to highlight below-chance levels in the revised figure (now Fig. 5 in the revised manuscript).

- There is no y-axis to index BIC levels on Figs 3 e-f

Thank you for this comment. In our previous Fig. 3, panels e and f were not aligned horizontally, which may have led to confusion about the (double) y-axis in these panels. We corrected this in the revision and improved our description of the double y-axes in the figure caption. Note that the panels show Rsq, which is inversely related to BIC, as to align with the interpretation of pxp in the same panels (i.e., higher levels indicate better fit).

Reviewer #2:
Remarks to the Author:

This study introduces a novel behavioral paradigm for the learning of abstract serial order. It then shows that reinforcement learning algorithms with asymmetric updating rules can provide an account of human performance in this paradigm. These are notable contributions to the field. There are some omissions and inconsistencies that should be addressed. The manner in which the data are reported is quite frustrating. The human data are simply reported as a summary statistic presumably reflecting average performance of all subjects over some time interval. There is little information about between-subject variation in learning rate or asymptotic performance. This makes it difficult for the reader to appreciate how well the various models capture important aspects of the data.

We wish to thank the reviewer for their positive evaluation of our manuscript and for the helpful comments.

General comments:

Partial feedback, as applied to transitive inference, appears to be a novel behavioral paradigm with some intriguing properties. It has been used before, but usually in a transfer paradigm and not a situation where subjects are simultaneously training on adjacent pairs and being tested on non-adjacent pairs. This has the benefit of allowing one to compare learning for adjacent and non-adjacent pairs in parallel. Unfortunately, the dynamics of learning (e.g. performance vs. trial number) are not reported for the human subjects. This is a significant omission for a paper about learning, and deprives the reader of being able to really appreciate the richness of the data.

We can only agree with this comment. In hindsight, the omission in our previous manuscript was a missed opportunity, given the nature of our paradigm. In the revision, we included a full new figure (Fig. 4 in the revised paper) detailing the dynamics of learning for non-adjacent (panels a-c) and adjacent pairs (panels d) over trials. The new figure provides additional validation of our model, and we are grateful that the reviewer encouraged it.

New Fig. 4

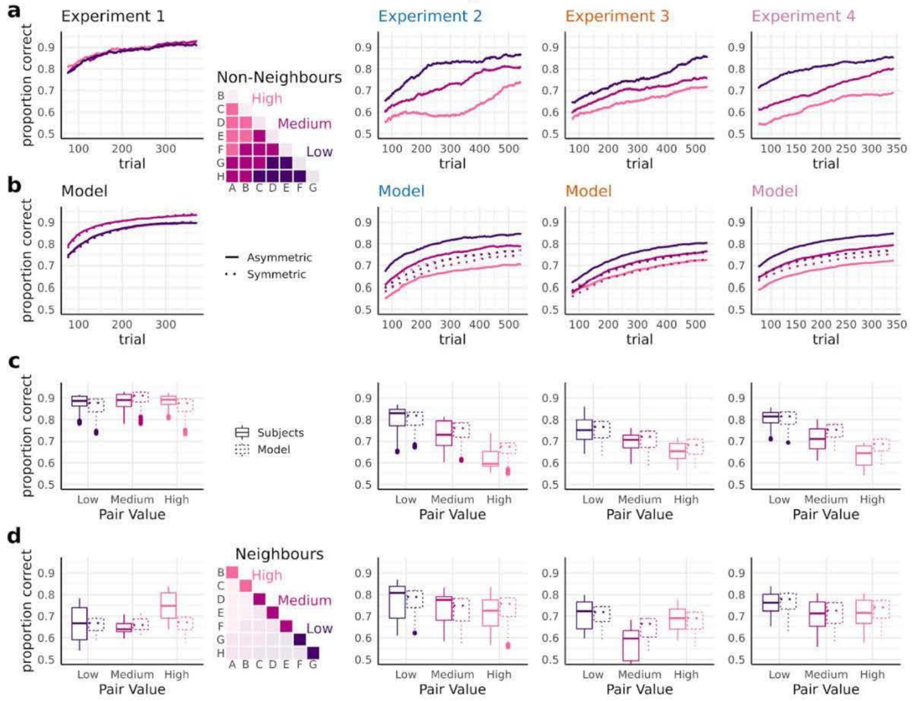

We designed the new figure to illustrate the match between model predictions and human data features with respect to asymmetric learning. Showing the data separately for low/medium/high valued pairs (which our models predict to systematically differ under asymmetric but not under symmetric learning), the new figure highlights the key empirical hallmark of asymmetric learning, including its time course. The figure also addresses additional reviewer comments as detailed further below.

Asymmetric Q-learning is a somewhat novel modeling approach. However, the issue was discussed in Jensen et al., 2019 (see reference below), which may deserve some mention.

Thank you for this comment. The learning asymmetry discussed in Jensen et al., 2019 (ref. [37] in the revised paper) between chosen vs. not chosen items is related, but not identical to the winner/loser asymmetries identified in our study. We address this point in detail in the revised

paper (see also our reply to Reviewer #1). Specifically, we performed direct model comparison between our winner/loser models and alternative models that allow for learning asymmetries between chosen/non-chosen items. Our winner/loser model fitted the data better than the alternative model, which validates our previous findings and interpretation (p 14, middle in the revised manuscript). We thank both reviewers for bringing up this point.

It would be worth discussing the issue of stability over time. In particular, the Q2 model does not appear to be asymptotically stable. Rather, it appears that all of the values would eventually saturate at 1.0 given enough trials. Q2, on the other hand, does appear to be stable. This is an important consideration for model fitting and selection. It would seem difficult to fit an unstable model without making strong assumptions about learning rates. Furthermore, if the values of all items in the model do indeed saturate, then it is hard to see how this could be a viable model of TI, as it would eventually lose the ability to support non-random choices between pairs of items.*

The reviewer raises a thoughtful point about the asymptotic stability of model Q2 under full feedback (Fig. 2c). To be precise, in the full-feedback context (and under the extreme asymmetry illustrated for comparison in Fig. 2c), model Q2 may predict values to become ever more compressed with infinite learning trials. This is an interesting observation, which may help understand the inferior performance of asymmetric learning under full- but not under partial feedback. We added a note about this in the revised manuscript (Fig. 2 caption).

However, as is now illustrated better in our new Fig. 4b (*left*), this property of Q2 does not seem to pose a major problem in the context of our experiments (with finite trial numbers, and partly with probabilistic feedback). In our Exp. 1, the basic shape of learning curves predicted by Q2 in fact strongly resembles that of Q1 (which is asymptotically stable even under full feedback). In response to the reviewer, we show below that this would hold even for a case of extreme asymmetry (which we did not observe empirically under full feedback). The plot below shows a Q2-predicted learning curve fitted to the human data (like in Fig. 4b left) while enforcing extreme asymmetry by restricting the learning rate for losers (α^{-}) to be 0 (solid line). As can be seen, even under this extreme asymmetry, the model would predict a reasonably shaped learning curve within the time horizon of our experiment (the x-axis shows a sliding average across all trials, as in Fig. 4a, *left*).

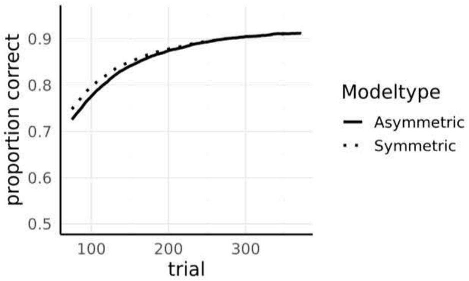

Importantly, however, we wish to clarify that in our paper, we do not present Q2 as a viable model of TI (i.e., under partial feedback). To the contrary, we explain in our simulations section (p xx) that the simple RL models (i.e., Q1/Q2) are ill-suited for TI under partial feedback (see also supplementary Movie M1). We made minor edits to this section for additional clarity. With regards to asymmetric vs. symmetric learning, the critical competitor model for model Q2* under partial feedback is in fact model Q1*, not Q2. Lastly, although we do not present Q2 as a model of TI, we wish to note that Q2 is asymptotically stable under partial feedback (where model Q2 outperforms Q1, but not Q2*; Fig. 3f). We wish to thank the reviewer for the thoughtful comment, which added to our understanding of how asymmetric learning can be beneficial under partial-feedback, but not under full-feedback.

The dynamics of learning are shown for the models, but not for the human data. Regarding the latter, it isn't clear if the data shown in Figs 3 and 4 are averaged over the entire session or reflect asymptotic performance. Likewise, it isn't clear if the models are fit to the entire time course of the data or to asymptotic performance. A figure that shows model and real performance as a function of trial number would help readers evaluate how well the models actually fit the data.

Our new Fig. 4 shows the dynamics of learning both for the human data and models (see also our reply to the reviewer's first comment above). Human data are always shown for the entire session, and models were always fitted to the entire time course. We made text edits to explain this better in the revised manuscript (e.g., Fig. 2a caption and p 30).

Specific comments

Figures – The color-coded half grids provide a general sense of the results and are fine for showing the model predictions (Fig. 2). However, for empirical data, this style of presentation makes it difficult for readers to see more subtle effects, make quantitative comparisons, or get a sense of the variability in the data. For figures 3 and 4, it might be advisable to use more conventional "box and whisker" style plotting.

We agree, and we have accommodated all these suggestions in our new Fig. 4. The figure also includes box-and-whisker style plots (panels c-d) which we agree are an important complement to our previous data presentation.

Likewise, the stacked histograms in Fig 4 (middle panel) are difficult to decipher. There seems to be enough space to separate these into 4 separate subpanels with one histogram each. This would be greatly appreciated.

We revised the figure (now Fig. 5) accordingly, now showing separate histograms for each experiment. In doing so, we realized that our previous illustration failed to convey an important aspect in comparing partial (Exp 2-4) and full feedback (Exp. 1) with respect to model-estimated asymmetry: whereas asymmetry improved the model fit (relative to symmetric models) in Exp. 2-4, it worsened the fit in Exp 1. To illustrate this in the revised figure, we

additionally color-coded the histograms, with stronger saturation indicating greater improvement in model fit (where it can be seen that even those Exp. 1 participants that might appear asymmetric in terms of Q2 parameter estimates were in fact better described by symmetric learning, Q1). We thank the reviewer for the valuable suggestion, which adds to the transparency and completeness of the visual results display.

New Fig. 5

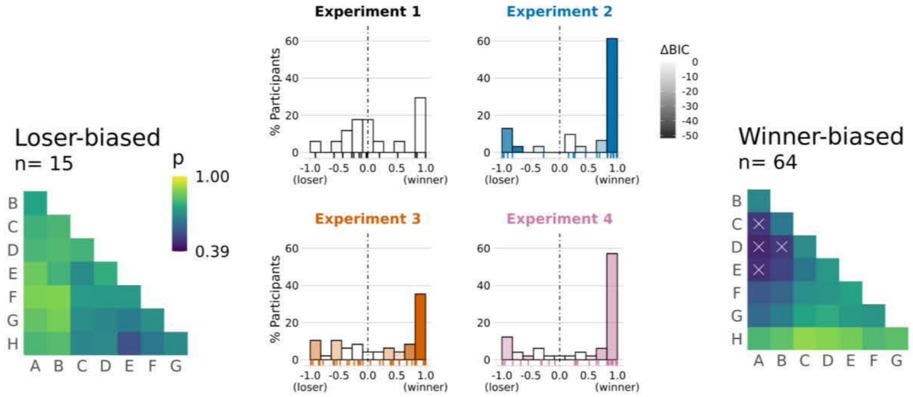

Models Q2 and Q2 make the strong prediction that performance should depend on absolute rank even for pairs that have the same symbolic distance. I.e., for distance=1, the pair AB should have the worst performance, while GH should have the best performance. This is borne out by the data only in the case of GH. All of the other pairs that have distance=1 appear to have about the same performance level. In other words, the strong vertical gradient shown for Q2 and Q2* in Fig. 2 does not appear to be supported by the data in Fig. 3. Furthermore, it is not clear why the model predictions for Q2* appear to be quite different in Fig. 2 vs. Fig. 3. Perhaps the authors could address the discrepancy.*

We believe that this point (which was also touched on by Reviewer #1) is resolved by our improvements in results figures, especially newly included Fig. 4d, which shows precisely the relevant data feature side-by-side with model predictions.

Crucially, our winning models do not predict a very strong gradient across distance=1 (i.e., neighbour) pairs in Fig. 3. This has two reasons. First, Fig. 3 (and new Fig. 4) show grand average data, where winner- and loser- focused individuals are combined; for comparison, see our new Fig. 5). The mean asymmetry in Fig. 3 and Fig. 4 is thus naturally less pronounced than in our Fig 2 simulations, where we illustrate the extreme case of zero learning for losers.

Second, even in the case of extreme asymmetry (cf. Fig. 2 and new Fig 5, left and right), the gradient across neighbour pairs will not be as pronounced when the winning model also

includes pair-level learning (+P; which was the case in the majority of our participants). To illustrate, we reran the critical simulation in Fig 2f (Q2*, here shown left, which shows the gradient mentioned by the reviewer) with additional pair-level learning (i.e., Q2*+P; here shown right):

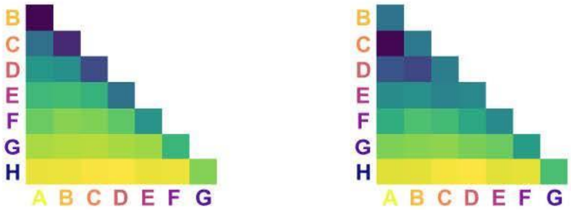

It can be seen that the gradient in question (i.e., at distance=1 along the diagonal) becomes more difficult to discern when pair-level learning (+P) is added (right panel; note that +P exclusively affects distance=1 pairs). In fact, the strength of the model-predicted gradient is well in line with that observed in our strongly asymmetric participants, which are illustrated in our new Figure 5.

As we now illustrate better also in our new Fig. 4, a much clearer empirical signature of value compression in our winning model is the gradient of accuracy across non-neighbouring pairs (i.e., distances >1), which is clearly borne out in the experimental data (Fig. 4, a-c).

We hope that together, these demonstrations illustrate clearly that the predictions from our models in fact align very well with the human observer data, even with respect to the specific data feature noted by the reviewer.

References

Jensen G, Terrace HS, Ferrera VP. Discovering Implied Serial Order Through Model-Free and Model-Based Learning. Front Neurosci. 2019 Aug 20;13:878. doi: 10.3389/fnins.2019.00878. PMID: 31481871; PMCID: PMC6710392.

We added this reference, which provides an excellent overview of previous work on transitive inference. Thank you very much.

We wish to thank both reviewers for the in-depth review of our manuscript and for the many valuable suggestions and comments, which helped us to substantially improve the paper. We are especially grateful to the reviewers and the editor for devoting their time and expertise during these difficult times of the pandemic.

FURTHER REVISIONS (independent of the reviewing process)

Performing additional code review during the revisions, we noticed that the protected exceedance probabilities (pxp) reported in our previous Fig. 3e-f were based on an outdated algorithm[1], which returned unprotected (rather than protected) exceedance probabilities. Thus, the numerical values were not fully accurate. In the revised paper, we updated the pxp values using the most current and accurate algorithm[2]. While the update led to changes in numerical values, it did not alter any of the results and conclusions from our previous model comparison. For transparency, we show below the pxp values (red bars, right y-axis) from the previous (top) compared to the updated (bottom) model comparisons in Fig. 3e-f.

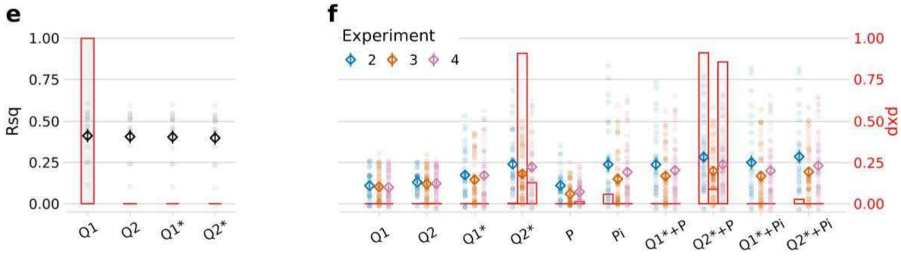

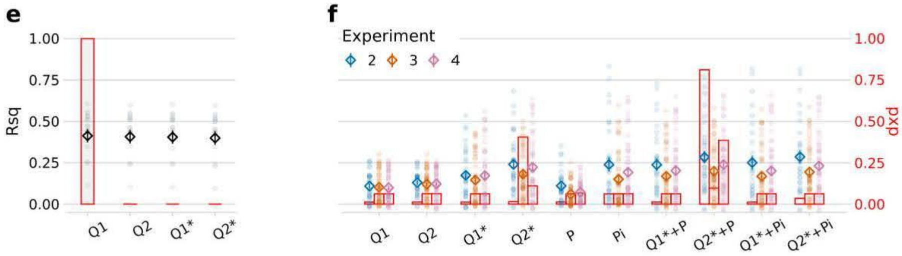

Please note that the updated pxp values (bottom panels) identify the exact same winning models in each experiment as before. Also note that our model comparisons based on BICs (cf. Rsq) are entirely unaffected by this revision, so that all our previous findings and conclusions stand unchanged.

However, the updated pxp values made us reconsider a design choice we had made in our previous Figures 3 and 4, where we had plotted the results of Q2*+P for Exp. 2 and 4 (where Q2*+P is unequivocally the best model both in pxp and in BIC/Rsq), but the results of Q2* for Exp. 3 (where Q2*+P was best fitting in terms of BIC/Rsq, but Q2* had a much higher pxp; note that the two models incorporate the same winner/loser asymmetries). In light of the new, more accurate pxp (where the evidence for Q2* in Exp. 3 is not as pronounced), we find this mixed illustration no longer ideal. In the revised figures, thus, we consistently show the results of Q2*+P (the best fitting model in each of Exp. 2-4) for each of the three experiments. We believe this also improves the overall accessibility of our results report (cf. comments by Reviewer #1). The adjustment entails a minor change in one of the numbers shown in Fig. 5

(previously Fig. 4; from 17 to 15), but no changes in any of the analysis outcomes and findings reported throughout the paper.

1. Stephan, K. E., Penny, W. D., Daunizeau, J., Moran, R. J. & Friston, K. J. Bayesian model selection for group studies. *NeuroImage* **46**, 1004–1017 (2009).

2. Rigoux, L., Stephan, K. E., Friston, K. J. & Daunizeau, J. Bayesian model selection for group studies — Revisited. *NeuroImage* **84**, 971–985 (2014).

Decision Letter, first revision:

Our ref: NATHUMBEHAV-210314663A

7th September 2021

Dear Bernhard,

Thank you once again for submitting your revised manuscript, entitled "Asymmetric learning facilitates human inference of transitive relations," and for your patience during the re-review process.

Your manuscript has now been evaluated by our referees, and in the light of their advice I am delighted to say that we can in principle offer to publish it.

Reviewer #1 included a couple of helpful suggestions for further improvements, which you may wish to incorporate.

Before we can issue formal acceptance, you must also revise your paper to ensure that it complies with our Guide to Authors at http://www.nature.com/nathumbehav/info/gta.

We are now performing detailed checks on your paper and will send you a checklist detailing our editorial and formatting requirements within two weeks. Please do not upload the final materials and make any revisions until you receive this additional information from us.

Among the list of comments will be the suggestion to change the title of your paper, to specify the type of learning, e.g., "Asymmetric reinforcement learning facilitates human inference of transitive relations".

Please do not hesitate to contact me if you have any questions.

Sincerely,

Marike

Marike Schiffer, PhD
Senior Editor
Nature Human Behaviour

Reviewer #1 (Remarks to the Author):

I thank the authors for their very constructive approach to this review process. I feel that most points raised (by me and the other reviewer) have been addressed satisfactorily. Notwithstanding a couple of very minor and cosmetic points (see below), I am happy to recommend this paper for publication, and congratulate the authors for a very nice study.

Minor:
- Regarding one of my previous point, and considering that the paper on transitive inferences seem to draw heavily from the reinforcement-learning literature, I would have appreciated a couple of explicit links/discussions in the paper (e.g. parallel between Actor-Critic and Pair-Level Learning,…)

- The asymmetric learning, although inferring efficiently the transitivity structure, also distort the absolute "values" that best/initially represented this transitivity structure (from -1 to 1) to a new relative-value scale (from 0/1 or -1/0). This raise the question on whether/how the relational structure can used in a generalization context, e.g. if one face, after learning, a new item with known absolute value (on the original scale -1/1). Maybe this could also be shortly discussed ?

Reviewer #2 (Remarks to the Author):

I appreciate the authors' thoughtful rebuttal and thorough revisions. All of my concerns have been addressed. I have no further comments.

Decision letter, final requests:

** Please ensure you delete the link to your author homepage in this e-mail if you wish to forward it to your co-authors. **

Our ref: NATHUMBEHAV-210314663A

30th September 2021

Dear Dr. Spitzer,

Thank you for your patience as we've prepared the guidelines for final submission of your Nature Human Behaviour manuscript, "Asymmetric learning facilitates human inference of transitive relations" (NATHUMBEHAV-210314663A). Please carefully follow the step-by-step instructions provided in the attached file, and add a response in each row of the table to indicate the changes that you have made. Please also check and comment on any additional marked-up edits we have proposed within

the text. Ensuring that each point is addressed will help to ensure that your revised manuscript can be swiftly handed over to our production team.

We would hope to receive your revised paper, with all of the requested files and forms within two-three weeks. Please get in contact with us if you anticipate delays.

When you upload your final materials, please include a point-by-point response to any remaining reviewer comments.

If you have not done so already, please alert us to any related manuscripts from your group that are under consideration or in press at other journals, or are being written up for submission to other journals (see: https://www.nature.com/nature-research/editorial-policies/plagiarism#policy-on-duplicate-publication for details).

Nature Human Behaviour offers a Transparent Peer Review option for new original research manuscripts submitted after December 1st, 2019. As part of this initiative, we encourage our authors to support increased transparency into the peer review process by agreeing to have the reviewer comments, author rebuttal letters, and editorial decision letters published as a Supplementary item. When you submit your final files please clearly state in your cover letter whether or not you would like to participate in this initiative. Please note that failure to state your preference will result in delays in accepting your manuscript for publication.

In recognition of the time and expertise our reviewers provide to Nature Human Behaviour's editorial process, we would like to formally acknowledge their contribution to the external peer review of your manuscript entitled "Asymmetric learning facilitates human inference of transitive relations". For those reviewers who give their assent, we will be publishing their names alongside the published article.

Cover suggestions

As you prepare your final files we encourage you to consider whether you have any images or illustrations that may be appropriate for use on the cover of Nature Human Behaviour.

Covers should be both aesthetically appealing and scientifically relevant, and should be supplied at the best quality available. Due to the prominence of these images, we do not generally select images featuring faces, children, text, graphs, schematic drawings, or collages on our covers.

We accept TIFF, JPEG, PNG or PSD file formats (a layered PSD file would be ideal), and the image should be at least 300ppi resolution (preferably 600-1200 ppi), in CMYK colour mode.

If your image is selected, we may also use it on the journal website as a banner image, and may need to make artistic alterations to fit our journal style.

Please submit your suggestions, clearly labeled, along with your final files. We'll be in touch if more information is needed.

ORCID

Non-corresponding authors do not have to link their ORCIDs but are encouraged to do so. Please note

that it will not be possible to add/modify ORCIDs at proof. Thus, please let your co-authors know that if they wish to have their ORCID added to the paper they must follow the procedure described in the following link prior to acceptance: https://www.springernature.com/gp/researchers/orcid/orcid-for-nature-research

Nature Human Behaviour has now transitioned to a unified Rights Collection system which will allow our Author Services team to quickly and easily collect the rights and permissions required to publish your work. Approximately 10 days after your paper is formally accepted, you will receive an email in providing you with a link to complete the grant of rights. If your paper is eligible for Open Access, our Author Services team will also be in touch regarding any additional information that may be required to arrange payment for your article. Please note that you will not receive your proofs until the publishing agreement has been received through our system.

Please note that <i>Nature Human Behaviour</i> is a Transformative Journal (TJ). Authors may publish their research with us through the traditional subscription access route or make their paper immediately open access through payment of an article-processing charge (APC). Authors will not be required to make a final decision about access to their article until it has been accepted. Find out more about Transformative Journals

<B>Authors may need to take specific actions to achieve compliance with funder and institutional open access mandates. For submissions from January 2021, if your research is supported by a funder that requires immediate open access (e.g. according to Plan S principles) then you should select the gold OA route, and we will direct you to the compliant route where possible. For authors selecting the subscription publication route our standard licensing terms will need to be accepted, including our self-archiving policies. Those standard licensing terms will supersede any other terms that the author or any third party may assert apply to any version of the manuscript.

For information regarding our different publishing models please see our Transformative Journals page. If you have any questions about costs, Open Access requirements, or our legal forms, please contact ASJournals@springernature.com.

Please use the following link for uploading these materials:
[REDACTED]

If you have any further questions, please feel free to contact me.

Best regards,
Chloe Knight
Editorial Assistant
Nature Human Behaviour

On behalf of

Marike

Marike Schiffer, PhD
Senior Editor
Nature Human Behaviour

Reviewer #1:
Remarks to the Author:
I thank the authors for their very constructive approach to this review process. I feel that most points raised (by me and the other reviewer) have been addressed satisfactorily. Notwithstanding a couple of very minor and cosmetic points (see below), I am happy to recommend this paper for publication, and congratulate the authors for a very nice study.

Minor:
- Regarding one of my previous point, and considering that the paper on transitive inferences seem to draw heavily from the reinforcement-learning literature, I would have appreciated a couple of explicit links/discussions in the paper (e.g. parallel between Actor-Critic and Pair-Level Learning,…)

- The asymmetric learning, although inferring efficiently the transitivity structure, also distort the absolute "values" that best/initially represented this transitivity structure (from -1 to 1) to a new relative-value scale (from 0/1 or -1/0). This raise the question on whether/how the relational structure can used in a generalization context, e.g. if one face, after learning, a new item with known absolute value (on the original scale -1/1). Maybe this could also be shortly discussed ?

Reviewer #2:
Remarks to the Author:
I appreciate the authors' thoughtful rebuttal and thorough revisions. All of my concerns have been addressed. I have no further comments.

Final Decision Letter:

Dear Bernhard,

We are pleased to inform you that your Article "Asymmetric reinforcement learning facilitates human inference of transitive relations", has now been accepted for publication in Nature Human Behaviour.

Please note that *Nature Human Behaviour* is a Transformative Journal (TJ). Authors whose manuscript was submitted on or after January 1st, 2021, may publish their research with us through the traditional subscription access route or make their paper immediately open access through payment of an article-processing charge (APC). Authors will not be required to make a final decision about access to their article until it has been accepted. IMPORTANT NOTE: Articles submitted before January 1st, 2021, are not eligible for Open Access publication. Find out more about Transformative Journals

Authors may need to take specific actions to achieve compliance with funder and institutional open access mandates. For submissions from January 2021, if your research is supported by a funder that requires immediate open access (e.g. according to Plan S principles) then you should select the gold OA route, and we will direct you to the compliant route where possible. For authors selecting the subscription publication route our standard licensing terms will need to be accepted, including our self-archiving policies. Those standard licensing terms will supersede any other terms that the author or any third party may assert apply to any version of the manuscript.

Before your manuscript is typeset, we will edit the text to ensure it is intelligible to our wide readership and conforms to house style. We look particularly carefully at the titles of all papers to ensure that they are relatively brief and understandable.

Once your manuscript is typeset and you have completed the appropriate grant of rights, you will receive a link to your electronic proof via email with a request to make any corrections within 48 hours. If, when you receive your proof, you cannot meet this deadline, please inform us at rjsproduction@springernature.com immediately. Once your paper has been scheduled for online publication, the Nature press office will be in touch to confirm the details.

Acceptance of your manuscript is conditional on all authors' agreement with our publication policies (see http://www.nature.com/nathumbehav/info/gta). In particular your manuscript must not be published elsewhere and there must be no announcement of the work to any media outlet until the publication date (the day on which it is uploaded onto our web site).

If you have posted a preprint on any preprint server, please ensure that the preprint details are updated with a publication reference, including the DOI and a URL to the published version of the article on the journal website.

An online order form for reprints of your paper is available at https://www.nature.com/reprints/author-reprints.html. All co-authors, authors' institutions and authors' funding agencies can order reprints using the form appropriate to their geographical region.

We welcome the submission of potential cover material (including a short caption of around 40 words) related to your manuscript; suggestions should be sent to Nature Human Behaviour as electronic files (the image should be 300 dpi at 210 x 297 mm in either TIFF or JPEG format). Please note that such pictures should be selected more for their aesthetic appeal than for their scientific content, and that colour images work better than black and white or grayscale images. Please do not try to design a cover with the Nature Human Behaviour logo etc., and please do not submit composites of images related to your work. I am sure you will understand that we cannot make any promise as to whether any of your suggestions might be selected for the cover of the journal.

You can now use a single sign-on for all your accounts, view the status of all your manuscript submissions and reviews, access usage statistics for your published articles and download a record of your refereeing activity for the Nature journals.

To assist our authors in disseminating their research to the broader community, our SharedIt initiative provides you with a unique shareable link that will allow anyone (with or without a subscription) to read the published article. Recipients of the link with a subscription will also be able to download and print the PDF.

As soon as your article is published, you will receive an automated email with your shareable link.

In approximately 10 business days you will receive an email with a link to choose the appropriate publishing options for your paper and our Author Services team will be in touch regarding any additional information that may be required.

You will not receive your proofs until the publishing agreement has been received through our system.

If you have any questions about our publishing options, costs, Open Access requirements, or our legal forms, please contact ASJournals@springernature.com

We look forward to publishing your paper.

All best

Marike

Marike Schiffer, PhD

Senior Editor

Nature Human Behaviour
